# Impact of HIV on Women in the United States^1^

**DOI:** 10.3201/eid1011.04-062308

**Published:** 2004-11

**Authors:** Hazel D. Dean, Lisa M. Lee, Melanie Thompson, Tracy Dannemiller

**Affiliations:** * Centers for Disease Control and Prevention, Atlanta, Georgia, USA;; † AIDS Research Consortium of Atlanta, Atlanta, Georgia, USA;; ‡ Lakeland, Florida, USA

**Keywords:** HIV, woman, United States

In the United States, AIDS was first reported in women in 1981 (*1*), and the percentage of AIDS cases in women has continued to increase, accounting for an estimated 26% of new AIDS diagnoses in 2002 (*2*). Since 1998, deaths among women with AIDS in the United States have remained stable at an estimated 4,000 (*2*).

## Epidemiologic Features of HIV in Women, United States

Data from 29 states with confidential name-based HIV reporting since 1998 were used to describe the status of HIV disease among women from 1999 through 2002. HIV diagnoses were defined as diagnoses of HIV infection regardless of AIDS diagnosis status. This diagnosis includes persons with a diagnosis of HIV infection only, HIV infection and later AIDS diagnosis, or concurrent diagnoses of HIV infection and AIDS.

From 1999 through 2002, an estimated 101,872 HIV diagnoses were reported from 29 states: 72,007 (70.7%) in men and 29,865 (29.3%) in women. Among women, 71.9% were non-Hispanic blacks, 18.2% were non-Hispanic whites, 8.4% were Hispanics, 0.6% were American Indian/Alaska Natives, and 0.4% were Asian/Pacific Islanders. The two principal modes of HIV exposure for women were heterosexual contact and injection drug use, accounting for 77.7% and 20.5% of diagnoses among women, respectively. Women were diagnosed with HIV at younger ages than men. For the 4-year period, 31.3% of women with HIV were in the 13- to 29-year age group compared with 19.9% of men in the same age group. HIV diagnosis rates were consistently higher among non-Hispanic black women compared with women from other racial and ethnic groups for all 4 years.

## Prevention Strategies for Women

In 2003, the Centers for Disease Control and Prevention (CDC) introduced the Advancing HIV Prevention (AHP) initiative (*3*). AHP aims to reduce barriers to early diagnosis of HIV infection, increase access to quality medical care and treatment, and provide ongoing prevention services for persons living with HIV. AHP incorporates four priority strategies: make voluntary HIV testing a routine part of medical care, implement new models for diagnosing HIV infections outside of the medical settings, prevent new infections by working with persons diagnosed with HIV and their partners, and decrease perinatal transmission.

## Clinical Care of Women with HIV

HIV-infected women may be at increased risk for medical problems and metabolic changes. Studies have shown that HIV-positive women were more likely to develop genital warts and cervical intraepithelial neoplasia (*4*) and were at increased risk for viral infections (*5*). According to one study, HIV-positive women were 80% more likely to be anemic than HIV-positive men (*6*). Compared with HIV-negative controls, women with HIV were more likely to have elevated triglycerides and insulin levels (*7*) and decreased bone mineral density (*8*).

Determining when to initiate antiretroviral therapy for HIV-infected women is based on CD4+ T cell count (*9*). Because no gender difference exists for initiating or applying antiretroviral drug regimens, the guidelines for treating women are the same as those for treating men. Overall, drug efficacy does not differ by gender in randomized clinical trials.

For many reasons, women with HIV may avoid HIV testing and care. Often, women may be stigmatized and endure discrimination because of their HIV status. Women are often the primary caregivers for other family members, which may lead to avoiding or delaying testing and care. Economic dependence on a spouse or significant other may also play a role in whether a woman seeks testing and care. Mistrust of the healthcare system may also exist. Depression or domestic violence may also affect a woman’s ability to seek needed care for HIV infection.

## Incorporating HIV Prevention into Medical Care

In 2003, CDC, the Health Resources and Services Administration, National Institutes of Health, and the HIV Medicine Association of the Infectious Diseases Society of America issued recommendations to assist clinicians in integrating HIV prevention into primary care for HIV-infected persons. Providers are encouraged to deliver brief prevention messages during primary care visits, screen for HIV risk behaviors and sexually transmitted disease, provide HIV behavioral risk-reduction messages, and facilitate partner notification and counseling (*10*).
